# Comparison of children's self-reports of depressive symptoms among different family interaction types in northern Taiwan

**DOI:** 10.1186/1471-2458-7-116

**Published:** 2007-06-20

**Authors:** Wen-chi Wu, Chi-Hsien Kao, Lee-Lan Yen, Tony Szu-Hsien Lee

**Affiliations:** 1 Center for Health Policy Research and Development. National Health Research institutes. No. 35, Keyan Road, Zhunan Town, Miaoli County 350, , R.O.C, Taiwan; 2 Institute of Health and Welfare Policy, National Yang-Ming University. No. 155, Sec. 2, Linong St., Beitou District, Taipei City 112, , R.O.C, Taiwan; 3 Institute of Health Policy and Management, College of Public Health, National Taiwan University. No.17 Xu-Zhou Road, Taipei 10020, , R.O.C, Taiwan; 4 Department of Health Promotion and Education, National Taiwan Normal University, Taiwan

## Abstract

**Background:**

Previous research has shown that family interactions are associated with depressive symptoms in children. However, detailed classifications of family interaction types have not been studied thoroughly. This study aims to understand the types of family interactions children experience and to identify the specific types of family interactions that are associated with a higher risk of depressive symptoms in children.

**Methods:**

Data used in the study was collected as part of the Child and Adolescent Behavior in Long term Evolution (CABLE) project in 2003. CABLE is a longitudinal cohort study that commenced in 2001 and collects data annually from children in Taipei city and Hsinchu county in northern Taiwan. The data analyzed in this study was that obtained from the sixth graders (aged 11 to 12 years old) in 2003. Of the 2,449 sixth graders, 51.2% were boys and 48.8% were girls. Factor analysis and cluster analysis were used to investigate the types of family interactions. One way ANOVA was used to establish the relationship between family interaction types and children's self-reports of depressive symptoms.

**Results:**

Based on the results of factor analysis, the latent factors for family interactions included supporting activities, psychological control, parental discipline, behavioral supervision, and family conflict. After conducting cluster analysis using factor scores, four types of family interactions were revealed: supervised (29.66%), disciplined (13.56%), nurtured (40.96%) and conflict (15.82%). Children from the disciplined or conflict families were more likely to report depressive symptoms. Children from the nurtured families were least likely to report depressive symptoms.

**Conclusion:**

Family interactions can be classified into four different types, which are related to children's self-reports of depressive symptoms. The creation of a family interaction environment that is beneficial for children's mental health is an important issue for health education and health promotion professionals.

## Background

According to the 2001 WHO World Health Report [1], the worldwide prevalence of depression is 3%, which equates to a total of 120,000,000 sufferers of depression. It is estimated that by 2020, depression will be the second cause of the Disability-Adjusted Life-Year (DALY) behind heart disease [2]. (The DALY is a quantitative indicator of burden of disease that reflects the total amount of healthy life lost to related causes during a period of time [3].) Depression has even been called the 'plague' of modern times by the mental health community [4]. People with symptoms of depression are more likely to have engaged in binge drinking and smoking, and are also more likely to have high blood pressure, high cholesterol, arthritis and asthma [5]. A US study has estimated that the health service costs for individuals with major depression are approximately 70% higher than for those without any depressive disorder [6]. Therefore, in addition to its influence on individual health, depression can increase overall health service costs.

A US review of depression related literature in the decade prior to 1996 showed that the onset of depressive symptoms is occurring at younger ages. Children and adolescents who were born at the end of the 20th century have a higher chance of developing mood disorders and of this continuing into adulthood. The review provided prevalence rates of depression ranging from 0.4–2.5% in children and 0.4–8.3% in adolescents. Furthermore, the lifetime prevalence rate of major depressive disorder in adolescents was estimated to range from 15% to 20%. [7]. In Taiwan, research conducted in 1998 measuring depressive symptoms in 1,434 seventh graders in Taipei found that 55.5% of adolescents reported having mild depressive symptoms (having 1–10 symptoms), 27.9% reported having more depressive symptoms (having 11–20 symptoms) and 9.9% reported serious depressive symptoms (having over 20 symptoms)[8]. The 1999 National Survey of Physical and Mental Health of Youth in Taiwan collected data from 3,487 adolescents aged 12–18 years old. It found that when the respondents encountered obstacles or stressful life events, 30.5% of them experienced depressive symptoms as their most frequent response out of eight possible emotional responses [9]. The variety of methods used to measure depressive symptoms and the different statistical methods used make it impossible to compare the rates of depression between studies. However, the high prevalence of symptoms found in the Taiwanese research indicates that childhood depression is still a very important issue for Taiwan.

Individuals who have depressive symptoms during childhood are more likely to develop psychiatric symptoms, aggression, poor adaptive function and low self-esteem during young adulthood [10,11]. Children with depression are more likely to suffer from separation anxiety, phobias, somatic complaints and behavioral problems [7]. They are also more likely to have difficulties at home, at school and with their peers and have less social adaptability [12]. Hence, depressive symptoms in children should be addressed early in order to prevent later psychiatric problems [10].

Previous research has investigated the relationship between family interactions and childhood depression [13-20]. In the United States, family environments of depressed children were found to be less rewarding, more aversive and more disengaged than those of non-depressed children [13], and adolescents with parents who frequently display anger or rejecting behaviors, and who rarely communicate with or encourage their children, are more likely to have depressive symptoms [14,15]. Children with a safer attachment relationship with their family and who receive more information, material goods, warmth and support from their family, are more likely to be able to use these resources to overcome stressful life events and as a result have better physical and mental health [16-19]. Similar results have been found in Taiwan where poor supervision, and strict, indirect and severe parenting styles have all been positively associated with depressive symptoms in adolescents [8]. In contrast, perceived positive messages from parents are negatively associated with depression in children [20].

A specific branch of research has focused on the associations between types of family interactions and children's depressive symptoms [14,21-25]. Stein et al [21] investigated the relationship between depression and family interactions in American children aged between 7 and 16 years. Scores for the two components of Care (which refers to the level of emotional expression, feeling of closeness and empathy between family members) and Protection (which refers to the level of parental control, supervision, and infantilization of children) were used to divide parent-child bonding into Affectionless-Control (low-care and high-protection), Weak (low-care and low-protection), Affective Constraint (high-care and high-protection), and Autonomy Supporting (high-care and low-protection). Stein's study showed that children with a high risk of depressive symptoms were more likely to report parental bonding as affectionless-control than low-risk children.

In the field of family system research, Olson et al [22] categorized family interactions according to three important components: Cohesion (refers to the strength of emotional connection in the family), Adaptability (refers to the ability of the family to change family norms, structure of rights and responsibilities, and role relationships), and Communication (refers to the ability of family members to exchange information). The components of cohesion and adaptability were then used to develop the Circumplex Model. This model proposes that family systems may range from extremely low cohesion to extremely high cohesion and from extremely low adaptability to extremely high adaptability. The families at the central level of cohesion and adaptability would function better than those who are at the highest and lowest levels of both dimensions. Lavee & Olson [23] further used these two dimensions to divide families into the following four types: Flexible-separated families (scored above the mean on the adaptability scale and below the mean on the cohesion scale), flexible-connected family (scored above the mean on both the cohesion and adaptability scales), structured-separated families (scored below the mean on both the cohesion and adaptability scales), and structured-connected families (scored below the mean on adaptability and above the mean on the cohesion scale). The results showed that people from flexible-separated and structured-separated families experienced more strain and poorer well-being than those from the other two groups.

Lamborn and Mounts [24] referred to Maccoby and Martin's conceptual framework [25] and divided parenting styles into four types (authoritative, authoritarian, indulgent, and neglectful) based on 2 dimensions: acceptance/involvement and strictness/supervision. The research revealed that adolescents who characterize their parents as neglectful (scores in the lowest tertiles on both dimensions) expressed more psychological problems then those who described their parents as authoritative.

In summary, the majority of previous research has divided family interactions into four types based on scores of two dimensions. However, in reality, the components of family interactions may be more complex and the range of types of family interactions could be more comprehensive if the components were measured more precisely. In this study, we collected data on a large number of items related to family interactions such as family activities, parental discipline and control, family support and family conflict. By including an extensive range of items we hoped to measure family interactions more completely. Our study aims to understand the types of family interactions of elementary school students and to further elucidate the relationship between family interaction types and childhood depressive symptoms.

## Methods

### Study participants

Data analyzed in this study was taken from the 2003 data collected as part of the Child and Adolescent Behavior in Long-term Evolution (CABLE) project [26]. CABLE commenced in 2001 with the random selection of 18 public elementary schools from Taipei city (representing a metropolitan area) and Hsinchu county (representing a rural area) in northern Taiwan. The CABLE cohort was established from the first and fourth grade students of the chosen schools and their parents [27]. Students whose parents agreed to their participation in the CABLE study filled out questionnaires in class. Data analyzed in our study was collected from the sixth grade student cohort in 2003 (which was the representative fourth grade sample in 2001). The age of the participants ranged from 11 to 12 years old. The original sample consisted of 2499 students, and after exclusion of incomplete questionnaires, a total of 2,449 participants were included in the analysis: 1,254 boys (51.2%) and 1,195 girls (48.8%). A total of 1,329 (54.3%) children were from Taipei city and 1,120 (45.7%) were from Hsinchu county. Approximately 57% of fathers had a high school level of education, and 28.9% had at least a college degree. About 65.9% of mothers had a high school level of education, and 18.9% had at least a college degree. In addition, 87.6% of parents were married.

### Study instrument and research variables

The questionnaire developed by the CABLE team for the sixth graders in 2003 included the core questions of previous years (including behavioral habits, psychological health, family interactions, peer relationships etc). Slight modifications were sometimes made to the annual questionnaires according to special issues in a particular year. We conducted a pilot study on June 2003 at two schools that were not part of this study. The results of this pilot study and the validity and reliability of questions were taken into consideration when modifying the questionnaire to produce the final version [27]. Data used in this study included information about the children's gender, children's self-reported depressive symptoms and children's perceptions of family interactions (see Appendix). Further explanations of the definition and measurement of children's self-reports of depressive symptoms and children's perceptions of family interactions are given below.

#### Depressive symptoms

Children's self-reports of depressive symptoms refer to the results of the CABLE Depression Scale filled out by the study participants. This scale was based on Kovac's [10,28] Children's Depression Inventory (CDI) and Faulstich et al's Center for Epidemiological Studies Depression Scale for Children (CES-DC) [29]. Participants rated how often in the past two weeks they experienced the following seven emotions: "Didn't feel like eating favorite foods", "Felt very sad", "Cried for no reason", "Found it hard to carry out tasks", "Felt frightened", "Didn't sleep well", and "Lacked motivation". Each item had a possible score of one to three points (1 = never, 2 = once or twice, 3 = many times). The scores from each of the seven items were added together to give an overall total score ranging from seven to twenty-one. Higher scores indicated that children reported more depressive symptoms. The Cronbach's α of the depressive symptom scale was 0.75. The results of exploratory factor analysis indicated that the seven items could be categorized into one factor.

#### Family interactions

The development of the CABLE Family interactions Scale was based on relevant literature [7,8,14-25] and also included some culturally specific questions. It included six aspects: family activities, parental discipline, parental support, psychological control, behavioral supervision and family conflict. Each item was scored from one to four (1 = never, 2 = one or two days, 3 = many days, 4 = every day). Family activities included activities that children performed with their parents during the past week such as talking, eating, doing housework, doing homework, playing at home, and going out somewhere. 'Parental discipline' referred to methods that children reported their parents used to punish them such as being grounded, banned from doing something they like, pocket money taken away, made to do housework, made to kneel, made to stand or physically punished. 'Parental support' referred to the level of positive support given to children by parents including encouragement, praise, consolation when upset, caring, listening, concern for school life, helping when needed and offering reasons when asking them to do something. 'Psychological control' referred to the reported level of parental control over children and was assessed by questions such as: "Do your parents make you feel they are always right?", "Do your parents often say to you 'Children don't understand things. Wait until you are older and then you will understand?", "Do your parents remind you about past mistakes when you are in trouble?, "Do your parents interrupt you when you are talking?", "Do your parents blame you for things that other people in the house have done?", and "When you and your parents disagree about something, are they not nice to you?". 'Behavioral supervision' referred to the reported level of understanding and control that parents have over children's behavior and was assessed by questions such as: "Do your parents know what you do in your free time?", "Do your parents know what you do after school?", "Do your parents know who you mostly hang out with?", and "Do your parents know how you spend your pocket money?". 'Family conflict' referred to any reported episodes of conflict between the child and other family members within the last month, and included quarrels or fighting with siblings, and quarrels with parents or other senior family members.

### Data management and statistical analysis

Data analysis was conducted using SPSS statistical software version 11.0. We used frequencies and percentages to express the distributions of depressive symptoms and we used means and standard deviations to express the characteristics of the total depressive symptom scores. To clarify the family interaction types of study participants, first we conducted factor analysis to analyze the latent factor structure of family interactions. Then cluster analysis was performed to group participants according to the factor scores for characteristics of family interactions. Finally, family interaction types were defined based on the results of one way ANOVA which showed differences in each factor between different clusters. In addition one way ANOVA was also used to establish the relationship between family interaction types and childhood depressive symptoms.

## Results

### Children's self-reports of depressive symptoms in study participants

As shown in Table 1, the average depression score in the study sample was 10.56. Girls (10.71) reported slightly higher scores for depressive symptoms than boys (10.42). Since the range of the depressive symptoms score was 7 to 21 points, the results indicate that the participants in this study reported only mild depressive symptoms. Generally, more than half of participants reported that they felt very sad (67.2%), found it hard to carry out tasks (54.4%) or lacked motivation (54.3%). Out of the seven depressive symptoms, girls were significantly more likely to report feeling sad, crying for no reason, and feeling frightened. Boys were more likely than girls to report finding it difficult to carry out tasks.

**Table 1:** Descriptive statistics for depressive symptoms in the study sample

Depressive symptoms	Never				Once or twice				Many times				Chi-square test
	boys		girls		boys		girls		boys		girls		
		
	n	(%)	n	(%)	n	(%)	n	(%)	n	(%)	n	(%)	
1. Didn't feel like eating	713	(56.9)	656	(54.9)	483	(38.5)	497	(41.6)	58	(4.6)	42	(3.5)	3.714
2. Felt very sad	457	(36.4)	344	(28.8)	676	(53.9)	689	(57.7)	121	(9.6)	161	(13.5)	20.280***
3. Cried for no reason	1056	(84.3)	876	(73.4)	166	(13.2)	262	(22.0)	31	(2.5)	55	(4.6)	43.555***
4. Found it hard to carry out tasks	546	(43.6)	570	(47.8)	556	(44.4)	531	(44.5)	151	(12.1)	92	(7.7)	13.953**
5. Felt frightened	968	(77.2)	832	(69.6)	250	(19.9)	309	(25.9)	36	(2.9)	54	(4.5)	18.692***
6. Didn't sleep well	774	(61.8)	726	(60.8)	376	(30.0)	391	(32.7)	103	(8.2)	78	(6.5)	3.910
7. Lacked motivation	596	(47.5)	526	(44.0)	529	(42.2)	556	(46.5)	129	(10.3)	113	(9.5)	4.678

	Whole sample				Boy				Girl				T-test
	
depressive symptoms score(7~21points)	n = 2441 mean = 10.56 SD = 2.66				n = 1251 mean = 10.42 SD = 2.58				n = 1190 mean = 10.71 SD = 2.73				-2.65**

**: p < 0.01; ***: p < 0.001

Note: The Kurtosis of the depressive symptoms score was 0.771, and the Skewness was 0.864.

### Types of children's perceived family interactions among study participants

#### Factor structure of children's perceived family interactions

Table 2 shows the results of the factor analysis of "Family interactions". The Bartlett's test of sphericity and Kaiser-Meyer-Olkin (KMO) were used to determine the appropriateness of the data set for factor analysis [30]. Bartlett's test of sphericity was evaluated for the factorability of the correlation matrix (i.e., to determine whether the items could be classified into categories). The significant Bartlett's test result (p < 0.001) indicated that the correlation matrix was significantly different from an identity matrix and appropriate for factor analysis (table 2). The KMO measure of sampling adequacy was 0.920, indicating that the correlation matrix had sufficient structure to result in a factorable solution [30]. Therefore, the 34 items under family interactions were suitable for factor analysis.

**Table 2:** Rotated factor structure of family interactions in the study sample

Family interactions	Factor 1 **Supporting activities**	Factor 2 **Psychological control**	Factor 3 **Parental discipline**	Factor 4 **Behavioral supervision**	Factor 5 **Family conflict**	Communalities
1. Parents praise you	**.804**	-.011	-.104	.065	-.043	.663
2. Parents comfort you	**.800**	-.074	-.095	.094	-.080	.670
3. Parents encourage you	**.792**	-.054	-.152	.066	-.060	.662
4. Parents help you solve problems	**.756**	-.045	-.103	.172	-.069	.619
5. Parents listen to what you say	**.711**	-.162	-.136	.135	-.112	.581
6. Parents care about what happens at school	**.674**	-.012	-.014	.196	-.086	.501
7. Parents look after you when you are sick	**.664**	.023	-.060	.142	-.030	.465
8. Parents tell you why they want you to do things	**.613**	.046	-.090	.152	-.082	.416
9. Parents talk with you	**.579**	-.126	-.017	.295	.060	.442
10. Parents play with you at home	**.502**	-.362	.153	.293	.096	.502
11. Parents take you on outings	**.466**	-.320	.143	.200	.162	.406
12. Parents do your homework with you	**.454**	-.308	.253	.290	.082	.455

13. Parents remind you about past mistakes when you are in trouble	-.162	**.634**	.303	.052	.051	.525
14. Parents interrupt you when you are talking	-.233	**.589**	.247	.028	.101	.473
15. Parents shout and swear at you	-.052	**.518**	.408	-.028	.096	.448
16. Parents blame you for things other people have done	-.267	**.492**	.267	-.010	.228	.437
17. Parents tell you that children don't understand things	.153	**.467**	.112	-.008	.133	.272
18. Parents make you feel that they are always right	.337	**.400**	.061	.045	-.063	.284
19. Parents are not nice to you when you disagree with them	-.183	**.389**	.286	-.013	.168	.295

20. Parents hit you	-.175	.331	**.590**	.022	.121	.504
21. Parents punish you by making you do housework or write lines	-.011	.068	**.587**	.018	.048	.352
22. Parents punish you by making you kneel or stand	-.086	.229	**.581**	-.009	.130	.415
23. Parents take away your pocket money or toys	-.051	.206	**.563**	-.066	-.060	.370
24. Parents ban you from doing something you like	-.051	.373	**.496**	-.012	-.014	.388
25. Parents lock you in a room	.004	.022	**.427**	-.079	.080	.195

26. Parents know what you do on the way home from school	.227	.066	-.187	**.676**	.023	.549
27. Parents know what you do in your free time	.315	-.015	-.170	**.660**	.021	.564
28. Parents know who you spend most of your time with	.300	.098	-.229	**.593**	-.019	.505
29. Parents know how you spend your pocket money	.147	.055	-.057	**.582**	-.114	.380
30. You do housework with your parents	.321	-.251	.252	**.452**	.009	.434
31. You eat with your parents	.326	-.131	.165	**.402**	-.009	.312

32. You have physical fights with your brothers and sisters	-.142	.016	.052	-.100	**.815**	.697
33. You have arguments with your brothers and sisters	-.108	.124	.044	-.122	**.805**	.692
34. You have arguments with your parents or other elders	-.077	.350	.055	-.228	**.399**	.343
Eigenvalue	8.0	3.6	1.6	1.4	1.3	
Post varimax explained variability (%)	16.6	7.6	7.3	6.9	5.0	
Accumulated explained variability (%)	16.6	24.2	31.5	38.4	43.5	
Cronbach's α	.900	.680	.740	.717	.657	

KMO = 0.920

Bartlett's test of sphericity: X^2 ^= 27766.51(df = 666, p < 0.001)

Principle component analysis was used to extract factors. The analysis was performed with an orthogonal (varimax) rotation and five factors were extracted based on eigenvalues (measures of variance) greater than 1. Each of these five factors explained 16.6%, 7.6%, 7.3%, 6.9% and 5.0% of variability respectively, which gave a combined total explanation of total variance of 43.5% (Table 2). The scale for measuring children's perceptions of family interactions in the CABLE study looked at the six aspects of parental support, family activities, psychological control, parental discipline, behavioral supervision, and family conflict. Apart from a small number of items, the results of factor analysis were mostly in agreement with the above six categories (Table 2). Factor one included twelve items, had a Cronbach's α value of 0.9 and was called 'Supportive activities'. Factor two included seven items, had a Cronbach's α value of 0.71 and was called 'Psychological control'. Factor three had six items, a Cronbach's α value of 0.70 and was called 'Parental discipline'. Factor four also included six items, had a Cronbach's α score of 0.72 and was called 'Behavioral supervision'. Factor five included three items, had a Cronbach's α of 0.66 and was called 'Family conflict'. The values of the associations between the five factor scores ranged from -0.005 to 0.009.

#### Clustering of children's perceived family interactions

Cluster analysis is used to classify subjects into different groups. Subjects in the same group share some common traits which means they have the smallest distance or largest similarity [31]. In this study, children with the greatest similarity in factor scores of children's perceived family interactions were classified into the same group. To identify the different types of children's perceived family interactions, a two-stage cluster analysis was carried out based on the above-calculated factor scores and with "children" as the unit of analysis. Firstly, Ward's hierarchical clustering method was used to choose the ideal number of clusters. When four groups were condensed into three groups, agglomerative coefficients increased from 11322.842 to 12253.122, and the greatest rate of change in the agglomerative coefficient was observed (5.95%–7.59%). As a result, it was decided to divide the study sample into four groups. K-means nonhierarchical cluster analysis was then used to divide the study sample into actual clusters. Scheffe's method was used to compare differences in the five factors among four clusters.

As shown in table 3, cluster one included 709 participants (29.66%), cluster two included 324 participants (13.56%), cluster three included 979 participants (40.96%), and cluster four included 378 participants (15.82%). The different clusters were given names based on which factor scores were high and low. The factor score for behavioral supervision in cluster one was 0.2130, which was higher than that for the other three clusters. The factor scores for the other interaction factors were lower and therefore this cluster was called the 'Supervised group'. The factor scores for psychological control (0.6512) and parental discipline (1.7356) were higher in cluster two and so this cluster was called the 'Disciplined group'. The factor scores for supportive activities (0.7472) and behavioral supervision (0.0813) were higher in cluster three and so this cluster was called the 'Nurtured group'. The factor scores for family conflict (1.6387) were higher in cluster four and so this cluster was called the 'Conflict group'.

**Table 3:** Comparison of factor and depressive symptom scores in the study sample according to the four family interactions types

	Means of Factor Scores and Depressive Symptom Scores					
			
Factors	Cluster 1 Supervised group (n = 709) (29.66%)	Cluster 2 Disciplined group (n = 324) (13.56%)	Cluster 3 Nurtured group (n = 979) (40.96%)	Cluster 4 Conflict group (n = 378) (15.82%)	F value	Scheffe's test
Supporting activities	-0.8974	-0.1913	**0.7472**	-0.0513	708.48***	3 > (2,4) > 1
Psychological control	0.0587	**0.6512**	-0.3404	0.2035	101.60***	2 > (1,4) > 3
Parental discipline	-0.4312	**1.7356**	-0.1528	-0.2821	756.54***	2 > 3 > 4 > 1
Behavioral supervision	**0.2130**	-0.1448	**0.0813**	-0.4454	42.69***	(1,3) > 2 > 4
Family conflict	-0.3735	-0.2199	-0.2810	**1.6387**	815.80***	4 > (1,2,3)

Depressive symptom score	10.56	11.94	9.61	11.79	113.297***	(2,4) > 1 > 3

***: p < 0.001

Note 1: The lowest F value was calculated from the natural logarithm of the depressive symptom score.

### Relationships between children's perceived family interaction types and children's self-reports of depressive symptoms

The bottom row of table 3 shows the relationship between children's perceptions of family interaction types and children's self-reported depressive symptom scores. Children with different types of family interactions had significantly different scores for depressive symptoms. The average score for depressive symptoms was highest in the Disciplined group (11.94), followed by the Conflict group (11.79) and the Supervised group (10.56). The average depressive symptom score was lowest in the Nurtured group (9.61). The children in this study reported only mild depressive symptoms. This is probably because the participants come from the general population.

## Discussion

Our results confirm that children's perceptions of family interactions have five underlying factors and that participants can be grouped into four different interaction types according to their factor scores. In addition, we were able to discern the types of family interactions that are associated with children's self-reports of depressive symptoms. Children from the disciplined or conflict groups were more likely to report depressive symptoms. On the other hand, children from the nurtured group were least likely to report depressive symptoms.

### Relationships between different types of family interactions and children's self-reports of depressive symptoms

The results found that children from the disciplined group were most likely to report depressive symptoms. In this group, factor scores for parental discipline and psychological control were much higher than in other groups. This means that children in disciplined families may be punished more frequently and may be more powerless than those from other groups. This result is similar to the previous finding that children from low affection, negative communication and high control families report more depressive symptoms [21,23,24]. The parents in disciplined families in Taiwan are comparable to the authoritarian families proposed by Lamborn [24]. These parents may be used to the traditional parenting style where children are raised in a strict or serious way [32,33] and where it is thought that severe methods of teaching, physical and oral punishment are a kind of general form of education [34]. A Taiwanese proverbs says "Children only have ears, and don't have a mouth". These kinds of statements may have an indirectly detrimental impact on a child's mental health and may lead children to think that they are incapable, unacceptable, or inadequate. Continual negative comments and inappropriate punishments from parents may be associated with the development of depression [35]. Therefore, it is important that Taiwanese parents pay more attention to the potential effect that negative statements and strict punishments have on children in the long term.

We found that children from the conflict group reported higher levels of depressive symptoms. Children's perceptions of family interactions in this group were characterized as having higher factor scores than the other groups for family conflict and lower scores for supervision. Few studies have included the concept of conflict in the family interaction structure. 'Conflict' in the context of this study is conceptualized as children answering back to their parents or elders or quarrelling with their siblings. It is similar to the daily hassles proposed by Chen's research involving 463 Taiwanese mothers and contributes to the mother's psychological distress [33]. Children in a family environment full of conflict have increased psychological burdens and chronic melancholia due to their powerlessness to change the situation [36].

Children from the supervised group reported lower levels of depressive symptoms than the previous two groups. This group was characterized by having the highest factor scores for behavioral supervision and the lowest factor scores for supportive activities, parental discipline and family conflict. Behavioral supervision was defined in this study as the level of parental knowledge about their child's daily life and included the level of parental participation and involvement in their child's life. A study from eastern Taiwan found that higher parental involvement was associated with lower childhood depression [20]. Our study also indicates that appropriate behavioral supervision is associated with fewer reported depressive symptoms. However, further research is still required to determine whether increasingly higher levels of behavioral supervision lead to increasingly lower levels of depression or if behavioral supervision of children can actually lead to an increase in childhood depression if it surpasses appropriate levels.

Children from the nurtured group reported the lowest levels of depressive symptoms. This group was characterized by having the highest frequency of supportive activities and the second highest frequency of behavioral supervision. This result demonstrates that when parents are more supportive of children or when positive interactions with family members are more common, children are less likely to report depressive symptoms. This result is supported by previous studies [18,19] and shows that children perceived positive support from parents and positive family interactions are related to fewer reported depressive symptoms.

### Gender differences in children's self-reports of depressive symptoms

As shown in Table 1, girls reported higher depressive symptom scores than boys. Moreover, girls were significantly more likely to report that they feel sad, cry for no reason, and feel frightened. Boys were more likely to report that they find something hard to carry out than girls. These findings are similar to the results of previous research [11,19,20,37,38]. This is probably because women are more likely to experience strain, to have a low sense of mastery, and to engage in ruminative coping [37]. It is also possible that girls are more vulnerable to stressful life events than boys, such as living in a nonsupportive, single-father household [19,38]. However, no conclusions can be reached in regards to the mechanism between gender and children's self-reports of depressive symptoms. Furthermore, the results of this study do not indicate that the effect of children's perceived family interaction types on childhood depressive symptoms is modified by gender.

### Study limitations

Our study had the following limitations. Firstly, the measures in the study were reported by children alone. However, although we cannot cross validate the answers with parents' or teachers' reports, the answers still represent the children's subjective perceptions of depressive symptoms and family interactions. Secondly, although an attempt was made to include a broad range of family interaction types, if the input items for factor analysis or cluster analysis are different, this may result in different kinds of grouping. Nonetheless, the results still show that family interaction types are not restricted to merely two components but rather can have multiple components. In addition, there is a definite relationship between types of children's perceived family interactions and children's self-reported depressive symptoms. However, as our study was cross-sectional in nature, we were unable to establish the direction of a possible causal association between family interactions and depressive symptoms. In other words, it may be possible that a child experiencing depressive symptoms will perceive their family environment and interactions as negative as opposed to negative family interactions leading to depressive symptoms. Finally, due to the sampling strategy used in this study, the results can be only generalized to public elementary school students in Taipei city and Hsinchu County in Taiwan.

## Conclusion

In conclusion, there are five latent components that can be extracted from children's perceived family interactions: supporting activities, psychological control, parental discipline, behavioral supervision, and family conflict. According to the factor scores of these five components, family interactions can be classified into four types: supervised (29.66%), disciplined (13.56%), nurtured (40.96%) and conflict (15.82%). Children's perceptions of family interaction types are related to children's self-reports of depressive symptoms. Children from disciplined families reported the most depressive symptoms and children from nurtured families reported the least depressive symptoms. Health and child care professionals should place more importance on the relationship between family interaction types and childhood depressive symptoms.

## Competing interests

The author(s) declare that they have no competing interests.

## Authors' contributions

WW has contributed to writing the paper and conducting statistical analyses. CHK reviewed the literature and completed the first draft. LLY has contributed to the study design and led the CABLE research team. TSHL provided suggestions for the discussion. All authors read and approved the final manuscript.

**Appendix 1:** Family interaction items and depressive symptoms

Interaction types and specific questions	Scale
**Family activities:**	
The following questions are about your experiences in your family.	
1. In the past week, did you talk with your parents?	1 = never
2. In the past week, did you eat with your parents?	2 = one or two days
3. In the past week, did you do housework with your parents?	3 = many days
4. In the past week, did your parents help you with your homework?	4 = every day
5. In the past week, did your parents play with you at home?	
6. In the past week, did your parents take you on outings?	
**Parental discipline:**	
When you misbehave, do your parents use the following ways to punish you?	
1. Lock you in a room?	1 = never
2. Ban you from doing something you like?	2 = once or twice
3. Take your pocket money or toys away?	3 = many times
4. Make you do housework or write lines?	4 = always
5. Shout or swear at you?	
6. Make you stand or kneel?	
7. Hit you?	
**Parental support:**	
The following questions are about your experiences of getting along with your parents lately.	
1. Do your parents encourage you when you are having trouble doing something?	1 = never
2. Do your parents praise you when you are a good boy/girl?	2 = once or twice
3. Do your parents comfort you when you are in a bad mood?	3 = many times
4. Do your parents look after you when you are sick?	4 = always
5. Do your parents listen to you when you have something to say?	
6. Do your parents care about what happens at school?	
7. Do your parents help you solve problems?	
8. Do your parents tell you why they want you to do something?	
**Psychological control:**	
The following questions are about your experiences of getting along with your parents lately.	
1. Do your parents make you feel that they are always right?	1 = never
2. Do your parents often say to you 'Children don't understand things. Wait until you are older and then you will understand?'	2 = once or twice
3. Do your parents remind you about past mistakes when you are in trouble?	3 = many times
4. Do your parents interrupt you when you are talking?	4 = always
5. Do your parents blame you for things that other people in the house have done?	
6. When you and your parents disagree about something, are they not nice to you?	
**Family conflict:**	
The following questions are about the relationships among your family members.	
1. In the past month, did you have arguments with your brothers and sisters?	1 = never
2. In the past month, did you have physical fights with your brothers and sisters?	2 = one or two days
3. In the past month, did you have arguments with your parents or other elders?	3 = many days
	4 = every day
**Behavioral supervision:**	
Please answer the following questions according to your experiences lately.	
1. Do your parents know what you do in your free time?	1 = don't know a thing
2. Do your parents know what you do after school?	2 = know a little bit
3. Do your parents know who you mostly hang out with?	3 = almost everything
4. Do your parents know how you spend your pocket money?	4 = definitely know
**Depressive symptoms:**	
The following questions are about your experiences in the past two weeks:	
1. In the past two weeks, did you not feel like eating even your favorite foods?	1 = never
2. In the past two weeks, did you feel sad or in a bad mood?	2 = once or twice
3. In the past two weeks, did you feel like crying for no reason?	3 = many times
4. In the past two weeks, did you find it difficult to carry out tasks?	
5. In the past two weeks, did you feel very frightened?	
6. In the past two weeks, did you have trouble sleeping?	
7. In the past two weeks, did you lack motivation to do things?	

## Pre-publication history

The pre-publication history for this paper can be accessed here:


